# LXR agonist increases apoE secretion from HepG2 spheroid, together with an increased production of VLDL and apoE-rich large HDL

**DOI:** 10.1186/1476-511X-10-134

**Published:** 2011-08-05

**Authors:** Makoto Kurano, Naoyuki Iso-O, Masumi Hara, Nobukazu Ishizaka, Kyoji Moriya, Kazuhiko Koike, Kazuhisa Tsukamoto

**Affiliations:** 1 Department of Metabolic Diseases, Graduate School of Medicine, The University of Tokyo, Tokyo 113-8655, Japan; 2 Department of Cardiovascular Medicine, Graduate School of Medicine, The University of Tokyo, Tokyo 113-8655, Japan; 3 Department of Infection Control and Prevention, Graduate School of Medicine, The University of Tokyo, Tokyo 113-8655, Japan; 4 Department of Gastroenterology, Graduate School of Medicine, The University of Tokyo, Tokyo 113-8655, Japan; 5 Department of Advanced Medical Science, The Institute of Medical Science, The University of Tokyo, Tokyo 108-8639, Japan; 6 Fourth Department of Internal Medicine, Mizonokuchi Hospital, Teikyo University School of Medicine, Kanagawa 213-8507, Japan; 7 Department of Metabolism, Diabetes and Nephrology, Preparatory Office for Aizu Medical Center, Fukushima Medical University, Fukushima 965-8555, Japan

**Keywords:** Spheroid HepG2 cells, LXR agonist, Apolipoprotein E, ApoE rich HDL, VLDL

## Abstract

**Background:**

The physiological regulation of hepatic apoE gene has not been clarified, although the expression of apoE in adipocytes and macrophages has been known to be regulated by LXR.

**Methods and Results:**

We investigated the effect of TO901317, a LXR agonist, on hepatic apoE production utilizing HepG2 cells cultured in spheroid form, known to be more differentiated than HepG2 cells in monolayer culture. Spheroid HepG2 cells were prepared in alginate-beads. The secretions of albumin, apoE and apoA-I from spheroid HepG2 cells were significantly increased compared to those from monolayer HepG2 cells, and these increases were accompanied by increased mRNA levels of apoE and apoA-I. Several nuclear receptors including LXRα also became abundant in nuclear fractions in spheroid HepG2 cells. Treatment with TO901317 significantly increased apoE protein secretion from spheroid HepG2 cells, which was also associated with the increased expression of apoE mRNA. Separation of the media with FPLC revealed that the production of apoE-rich large HDL particles were enhanced even at low concentration of TO901317, and at higher concentration of TO901317, production of VLDL particles increased as well.

**Conclusions:**

LXR activation enhanced the expression of hepatic apoE, together with the alteration of lipoprotein particles produced from the differentiated hepatocyte-derived cells. HepG2 spheroids might serve as a good model of well-differentiated human hepatocytes for future investigations of hepatic lipid metabolism.

## Background

Apolipoprotein E (apoE), a 34-kD glycoprotein produced mainly by hepatocytes and also secreted from several cells including macrophages and adipocytes, plays a crucial role in lipoprotein metabolism and atherosclerosis. It mediates the cellular uptake of several classes of lipoproteins by acting as a ligand for the chylomicron remnant receptor, the VLDL receptor, LDL receptor and the LDL receptor-related protein (LRP). ApoE produced by macrophages and those accessing macrophages from the bloodstream facilitate the reverse cholesterol transport by promoting the formation and maturation of HDL particles [1,2]. In addition to these functions, apoE produced in hepatocytes enhances the production of VLDL particles [3]. The increased production of hepatic VLDL particles, a phenomenon observed in insulin-resistant patients or some primary hyperlipidemia subjects, leads to the accumulation of atherogenic lipoproteins in the circulation resulting in the aggravation of atherosclerosis.

The genetic regulation of the apoE gene has been pursued extensively. Taylor et al has identified two hepatic enhancer elements located far-downstream of the apoE gene, and clarified the regions critical to the baseline expression of the apoE gene [4,5]. They also identified the duplicated downstream enhancer elements termed multienhancers (ME.1 and ME.2), and demonstrated that these elements are crucial for apoE expression in macrophages and adipocytes [6]. In addition, Laffitte et al elegantly clarified that the nuclear receptor liver × receptor (LXR) regulates apoE expression in adipocytes and macrophages through direct interaction of the LXR response element found in both ME.1 and ME.2 [7].

In spite of these extensive analyses on apoE gene regulation, physiological factors which affect gene regulation of apoE in the liver have not been elucidated so far. Previous in vivo studies utilizing guinea pig [8] and cebus monkey [9] have shown that cholesterol feeding to these animals resulted in the up-regulation of apoE gene in the liver, raising the possibility that the accumulation of cholesterol in hepatocytes would affect hepatic up-regulation of the apoE gene. In addition, investigation in mice also indicated the up-regulation of hepatic apoE gene by cholesterol feeding [10]. However, the contribution of LXR in the regulation of murine hepatic apoE was not demonstrated [7,10]. Furthermore, no study has clarified the role of LXR in the regulation of hepatic apoE gene in human-derived hepatocytes or hepatic cell lines, with only one exception which utilized artificial reporter gene construct, in which ME.1 or ME.2 was placed just before the -890 to +93 apoE promoter [7].

As for the model of human hepatocytes, HepG2 cells have been widely used for in vitro experiments; however HepG2 cells grown in monolayer form on a culture plate are different from the in vivo hepatocytes which exist in three dimensional form in the liver, and would not completely reflect the physiological functions of hepatocytes. HepG2 cells in spheroid culture, which grow in three dimensional form after being encapsulated in alginate beads [11], have been shown to be more differentiated than HepG2 cells cultured in monolayer form; the cells proliferating in alginate beads form cell-cell contact with each other, and normal hepato-cellular junctional complexes including canaliculi with microvilli are constructed [11]. In consequence, the production of several proteins and the detoxificatory functions [11] as well as the production of cholesterol and triglycerides [12] increased significantly in HepG2 cells in spheroid culture compared to those in monolayer culture.

In this report, we first compared the production of several apolipoproteins from HepG2 cells in spheroid culture with those in monolayer culture. Next, we examined the effect of TO901317, a synthetic LXR ligand, on the secretion of apoE as well as lipoproteins with HepG2 cells cultured both in three-dimensional form and in monolayer form.

## Results

### HepG2 cells cultured in spheroid form (S-Hep) secreted more albumin and apolipoproteins than HepG2 cells cultured in monolayer (M-Hep)

**Figure 1:**
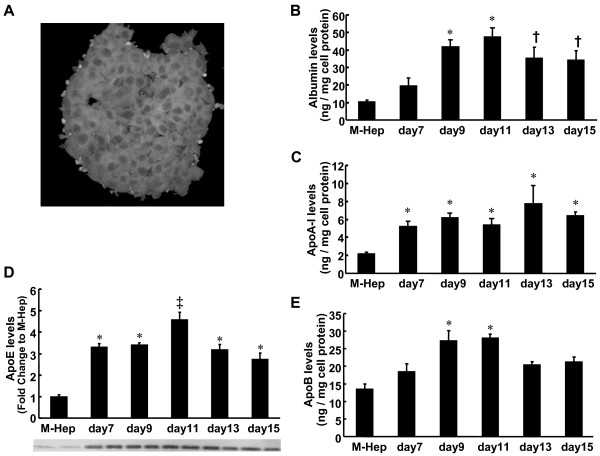
**Differences in albumin and apolipoproteins secretion between monolayer and spheroid HepG2 cells**. (A) Representative appearance of HepG2 spheroids on Day 11. HepG2 spheroids were fixed with 10% formalin solution, and examined with confocal microscopy. Briefly, from a single HepG2 cell, cells proliferate and form a spheroidal cell-cluster in an aliginate bead. (B-E) Time-course changes in the secretion of albumin (B), apoA-I (C), apoE (D), and apoB (E) from S-Hep cells. S-Hep (spheroid HepG2 cells) were prepared and grown in alginate beads as described in Materials and Methods, and the protein levels in the media on different time points after the beginning of the culture were measured. X axes represent the days after the beginning of S-Hep culture. Data are mean ± SEM (n = 4). *: P < 0.01 compared with M-Hep; †: P < 0.03 compared with M-Hep; ‡: P < 0.01 compared with M-Hep and P < 0.03 with S-Hep on the other time points.

**Figure 2:**
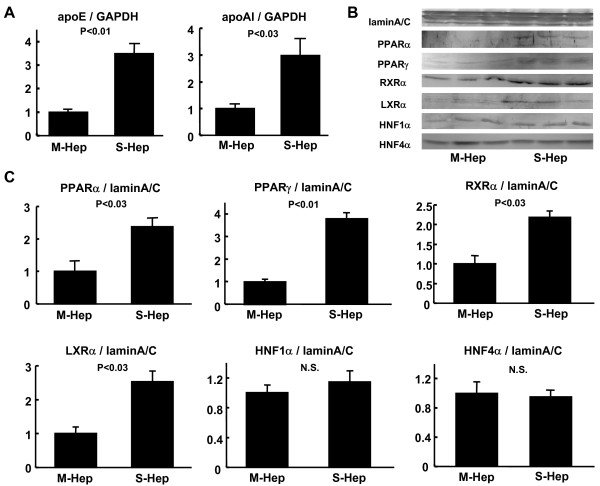
**Differences in apolipoprotein mRNA and nuclear receptor protein levels among M-Hep and S-Hep**. (A) Analyses of mRNA levels of apoE and apoA-I with quantitative RT-PCR (n = 4). GAPDH mRNA was used as the endogenous control for the expression levels of the gene of interest. (B, C) Western blot analyses were performed with the proteins prepared from nuclear fractions (Panel B), and nuclear protein levels were quantified with Image J utilizing lamin A/C as the internal control (Panel C) (n = 3). Y-axes in panels A and C represent ratios to the data of M-Hep.

HepG2 cells cultured in spheroid form grew in three dimensional form (Figure 1A). To validate the differentiation of HepG2 cells prepared as spheroids in our procedure, we first examined the time-course changes in the secretion of albumin. As shown in Figure 1B, the secretion of albumin from S-Hep was enhanced; the highest secretion level was observed on Day 11, reaching as high as 4.5-fold compared to M-Hep, which was concordant with the previous report [11]. The secretions of apolipoprotein A-I (apoA-I), apoE, and apolipoprotein B (apoB) did also increase in S-Hep, and the time-course changes in their levels were almost the same as those found with albumin (Figure 1C-E). The mRNA levels of apoA-I and apoE on Day 11 of S-Hep revealed a 3-fold and 3.5-fold increase compared to M-Hep (Figure 2A). Because the levels of albumin and apoE secretions were highest on Day 11, for the subsequent experiments, we utilized S-Hep on Day 11.

### PPAR-α, PPAR-γ, LXR-α, RXR-α were more abundant in the nuclear fractions of S-Hep than in those of M-Hep

In order to elucidate whether the up-regulation of the genes of apolipoproteins in S-Hep were associated with changes in the nuclear receptors, we next examined the nuclear protein levels in S-Hep in comparison with M-Hep. As shown in Figure 2B and 2C, the Western blot analyses of the proteins prepared from nuclear fractions revealed that peroxisome proliferator-activated receptor (PPAR)-α, LXR-α and retinoid × receptor (RXR)-α were more abundant in S-Hep than M-Hep. We did not observe differences in nuclear protein levels of hepatocyte nuclear factor (HNF)1-α and HNF4-α between S-Hep and M-Hep, however, PPAR-γ was also increased in S-Hep. This result suggested that the state of differentiation of hepatic derived cells would affect the expressions of several proteins associated with lipid metabolism at the level of DNA transcription.

### TO901317 increased apoE secretion and suppressed apoA-I secretion from HepG2 spheroids more evidently than monolayer HepG2 cells

**Figure 3:**
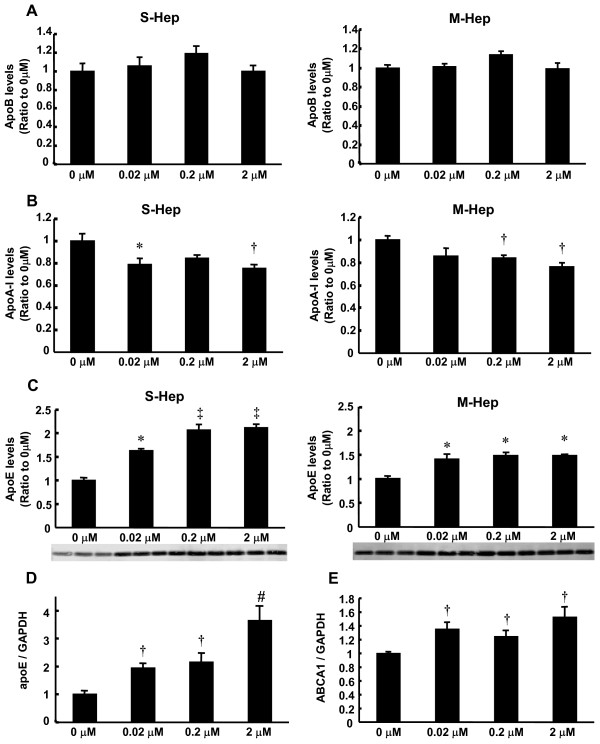
**Effects of TO901317 treatment on the secretion and expression of apolipoproteins. **(A-C) S-Hep and M-Hep were incubated with different concentration of TO901317, and the levels of apoipoproteins secreted in the medium were analyzed. Panel A, B and C represented apoB, apoA-I and apoE levels, respectively. (D, E) S-Hep were incubated with different concentration of TO901317, and mRNA levels of apoE (D) and ABCA1 (E) were analyzed. Data are mean ± SEM (n = 4). X-axes represent the concentration of TO901317. *: P < 0.01 compared with 0 μM, †: P < 0.05 with 0 μM, ‡: P < 0.01 with 0 and 0.02 μM, #: P < 0.03 with 0 and 0.02 μM.

The increased nuclear level of LXRα in S-Hep together with the increased secretion of apoE from S-Hep prompted us to evaluate the effect of LXRα agonist on the secretion of apoE, because LXRα has been identified as a critical factor for the regulation of apoE in macrophages and adipocytes. Thus we next examined the effect of TO901317 (TO), a synthetic LXRα agonist, on the apolipoproteins' secretion from S-Hep as well as M-Hep. As was shown in Figure 3A, the incubation of cells with TO did not alter the levels of apoB secretion in both S-Hep and M-Hep. The secretion of apoA-I was decreased in both forms of HepG2 cells when the cells were incubated with TO, which was concordant with the previous finding by Huuskonen et al (Figure 3B) [13]. On the other hand, apoE secretion was enhanced not only in S-Hep but also in M-Hep with the incubation of cells with TO (Figure 3C). The induction of apoE in M-Hep plateaued at 0.02 μM TO, while the dose-dependent increase in apoE secretion from S-Hep was observed up to 0.2 μM. In addition, this incremental apoE secretion was more prominent in S-Hep, reaching almost twice the level of cells without TO.

### TO901317 increased apoE and ABCA1 mRNA levels in HepG2 spheroids

In order to evaluate whether the increased secretion of apoE from S-Hep treated with TO was associated with the upregulation of the apoE gene, we examined the levels of apoE mRNA as well as ATP binding cassette transporter (ABC) A1 mRNA with quantitative real time PCR analyses. As shown in Figure 3D, the mRNA levels of apoE were significantly elevated by TO treatment, indicating that LXRα activated with TO would have increased the transcription of apoE in S-Hep. ABCA1, which is regulated by LXR, was also upregulated by TO901317, although the levels of the induction were less than those observed with apoE (Figure 3E).

### Distribution of apoE secreted from S-Hep among lipoproteins

**Figure 4:**
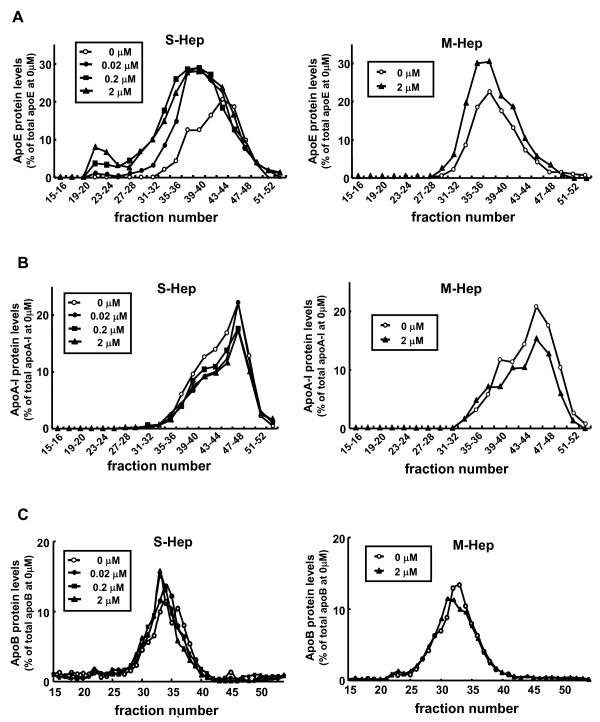
**Effects of TO901317 treatment on the distribution of apolipoprotein among lipoproteins**. Distribution of apoE (A), apoA-I (B), and apoB (C) on lipoprotein particles in the medium harvested from S-Hep (left) and M-Hep (right). Following the fractionation of the media with FPLC depending on the size of lipoproteins, fractionated samples were subjected to Western blot analyses or protein measurement with ELISA kits. White circles, black circles, squares and triangles represent the results from the medium of cells incubated with 0 μM, 0.02 μM, 0.2 μM and 2 μM of TO901317, respectively.

**Figure 5:**
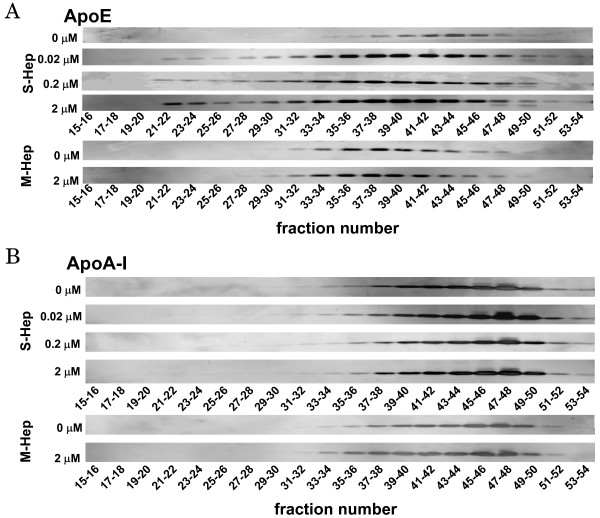
**Western blot analyses on the distribution of apoE and apoA-I proteins among lipoprotein classes**. After the incubation of S-Hep and M-Hep with TO901317 at the concentrations indicated, the media were fractionated with FPLC and the fractionated samples were subjected to Western blot analyses. Panel A and B represent the distributions of apoE and apoA-I, respectively.

**Figure 6:**
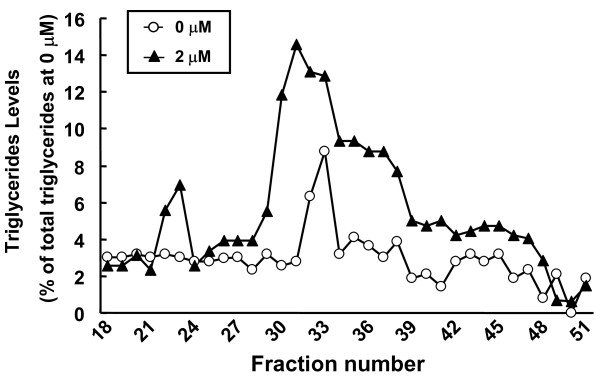
**Analyses of triglyceride levels in the FPLC-fractionated samples**. FPLC fractions obtained from S-Hep with or without 2 μM TO were subjected to the measurement of triglyceride (TG) levels with enzymatic method. White circles and triangles represent the results from the medium of cells incubated with 0 μM and 2 μM of TO901317, respectively.

As shown in the above experiments, we confirmed that the secretion of apoE was enhanced in S-Hep compared to M-Hep, and treatment of HepG2 cells with TO resulted in the augmentation of apoE secretion from HepG2 cells. We next analyzed the distributions of apoE, together with those of apoB and apoA-I, among lipoproteins after fractionating the media with fast protein liquid chromatography (FPLC) which separated lipoproteins depending on their sizes. The results are shown in Figure 4 and 5: the fractions 21 - 26 corresponded to VLDL, fractions 29 - 37 to LDL, and fractions 38 - 48 to HDL based on the analysis of human plasma (data not shown). Both in S-Hep and M-Hep, apoA-I distribution was noted almost exclusively on fractions corresponding to HDL fractions, especially small HDL fractions; this distribution was not altered even with TO treatment. ApoB proteins were detected in fractions relevant to VLDL and LDL fractions, although the apoBs found in VLDL were scarce. In addition, no difference was observed in the distribution patterns of apoB between S-Hep and M-Hep, and treatment with TO did not alter these patterns. In contrast, unlike the findings of apoA-I and apoB, the distribution of apoE on lipoproteins was affected not only by the methods of the culture but also by the treatment with TO. ApoE secreted from M-Hep, regardless of the treatment with TO, were detected in the fractions spanning between those of LDL and HDL, suggesting its distribution on large HDL fractions; in addition, no apoE band was found in VLDL fractions even by the treatment with TO. On the other hand, the culture of HepG2 cells in spheroidal form rendered the apoE protein to reside on normal-sized HDL particles. Interestingly, treatment of S-Hep with TO not only increased the amount of secreted apoE incrementally with the increment of the dose of TO, but also rendered apoE to distribute on fractions larger than normal HDL. Based on the observation that neither apoA-I nor apoB was detected in these lipoprotein fractions, these fractions were assumed to be large apoE rich HDL. Furthermore, TO treatment of S-Hep resulted in the appearance of apoE on VLDL fractions; the amount of apoE on VLDL increased incrementally when the concentration of TO was increased up to 2 μM. In addition, as shown in Figure 6, treatment of S-Hep with 2 μM TO resulted in the increased triglycerides levels in the fractions where apoE protein increased with TO treatment. These results indicated that the TO treatment not merely resulted in the increased apoE protein levels in VLDL and large HDL fractions, but also resulted in the increased particle numbers and/or the enrichment of lipid content of VLDL and large HDL particles.

## Discussion

Utilization of primary hepatocytes or hepatocyte-derived cell lines for in vitro experiments has helped us understand the lipid metabolism in the liver. However, it has been known that even the primary hepatocytes, when cultured in monolayer form, lose their differentiated, physiological functions quickly probably due to the loss of its three dimensional in vivo conformation in the experimental setting. In vivo experiments with rodents have also enabled us to elucidate the lipid metabolism in the liver; however, rodents might have been known to possess different properties from humans in lipid metabolism. Thus, in this study, in order to overcome these obstacles, we utilized 3-D spheroid culture system utilizing alginate beads, which has proven to be an easily-manipulative tactic to reproduce hepatocytes or hepatocytes-derived cells similar to their natural, differentiated in vivo counterparts.

Concordant with the previous observation by Khalil et al. [11], we were able to demonstrate that the culture of HepG2 cells in spheroid form resulted in enhanced albumin secretion compared to that in monolayer form, indicating that S-Hep utilized in this study had been more differentiated than M-Hep. The secretions of apoE, apoA-I, and apoB were also enhanced in S-Hep, and the nuclear protein contents of PPAR-α, PPAR-γ, LXR-α and RXR-α were increased in S-Hep, indicating that both of which are the features of differentiated hepatocytes or hepatocyte-derived cells. Furthermore, activation of LXR with TO901317 treatment of S-Hep resulted in the increased secretion of apoE protein which was accompanied by the up-regulation of apoE mRNA levels. Taking into account the previous study not showing a significant up-regulation of apoE gene with M-Hep treated with LXR activation [7], we assume that the differentiation of cells would be important to clearly examine the regulation of apoE gene in hepatocytes or hepatocyte-derived cells with LXR agonist, which is the case for macrophages and adipocytes [7].

The physiological regulation of apoE gene in the liver has so far not been clarified, although the baseline expression has been known to be controlled by distal hepatic enhancer elements [4,5] as well as the proximal promoter region to which TR4 orphan nuclear receptor binds [14]. Laffitte et al suggested that apoE enhancers and promoters containing LXRE would be important for the activation of apoE promoter in M-Hep; however, administration of LXR agonist in mice revealed a slight but non-significant role of LXR in the regulation of apoE in vivo [7]. Their observation does not depart at all from our present study, considering that murine lipid metabolism differs from that of humans in several respects. It is also plausible that factors other than LXR would regulate hepatic apoE gene in mice and probably in humans, considering that the degree of up-regulation of apoE gene observed in our study is less than those found in adipocytes and macrophages [7]. Further studies are needed to clarify these factors, and we believe that utilization of S-Hep would enable us to elucidate these factors.

In this study, we also found that the increased apoE secretion from S-Hep resulted in the alteration of lipoprotein classes produced from the cells. One of the aspects of this alteration is the increased production of VLDL particles. In the state of hepatic steatosis, not only triglycerides but also cholesterol accumulate in hepatocytes [15], and the oxidative stress which increases in hepatic steatosis [16] transforms the cholesterol to oxysterol, which is a natural ligand for LXR. Because the production of apoE is one of the important determinants for the secretion of VLDL or VLDL-TG from the liver [3], the upregulation of apoE gene together with the increased production of triglycerides [17,18] by LXR activation facilitates the production of VLDL particles, resulting in the atherogenic lipid profile of the metabolic syndrome.

The other interesting finding in the alteration of lipoprotein production from S-Hep with TO901317 treatment was the increased production of large HDL particles containing apoE. Treatment with TO901317 prevented atherosclerosis in various mouse models [19-21], and increased apoE-rich HDL particles in C57BL6 mice [22,23]. These reagent effects have been attributed to the enhanced reverse cholesterol transport from macrophages [24] through the up-regulation of several key macrophagic proteins such as ABCA1 and apoE [25,26]. However, in this study, we did indicate that apoE-rich large HDL particles were also produced from the differentiated hepatocyte-derived cells, and that the induction was more pronounced with increasing increments of LXR activation. Although apoE-rich HDL has been speculated to play a role in delivering cholesterol to hormone-producing tissues such as adrenal tissues [27,28], several lines of study have indicated that apoE-rich HDL would also play an important role in reverse cholesterol transport; apoE-rich HDL is mainly contained in large HDL and large HDL has been demonstrated to extract cholesterol from macrophages [29]. It was also suggested that apoE-containing HDL efficiently enhanced cholesterol efflux [2,30]. Thus the increased production of apoE-rich large HDL particles from differentiated hepatocytes induced by LXR activation might have a role in the protection against atherosclerosis.

## Conclusions

In summary, by utilizing the differentiated spheroid HepG2 cells, for the first time we were able to clearly demonstrate that LXR activation resulted in the up-regulation of human hepatic apoE, which also enhanced the production of VLDL particles and large apoE rich HDL particles. In future studies, investigation using HepG2 spheroids as surrogates to well-differentiated human hepatocytes would serve well as a model to precisely understand lipid metabolism in the liver.

## Methods

### Cell Culture and Experimental Protocol

HepG2 cells, purchased from American Type Culture Collection (ATCC, Manassas, VA), were cultured and maintained in DMEM (Sigma-Aldrich Co. St. Louis, MO) supplemented with 10% fetal bovine serum (FBS, Gibco BRL, Eggstein, Germany) and 1% penicillin/streptomycin (Gibco). For the experiment with M-Hep without TO (Sigma-Aldrich), 24 hours prior to the harvest of medium and cells, the medium was replaced with FBS-free medium to eliminate the plasma proteins derived from FBS in the medium. In the experimentation of M-Hep with TO, the medium was exchanged for that containing various concentrations of TO dissolved in DMSO at the cell confluency of around 70%. Two days later, the medium was replaced with the FBS-free medium containing the same concentration of TO, and cells were incubated for another 24 hours prior to the analysis. The collected cells were suspended in RIPA buffer (Santa Cruz Biotechnology, Santa Cruz, CA) for further analysis. The protein levels of the cell lysates were measured with Lowry methods (BioRad, Hercules, CA.) according to the manufacturer's protocol.

S-Hep were prepared following the methods described previously [11,12] with some modification. Briefly, HepG2 cells cultured in monolayer were detached completely with Trypsin-EDTA (Gibco) and suspended in α-MEM (Gibco) containing 10% FBS at the concentration of 0.5 × 10^6^/mL. The medium containing HepG2 cells was mixed with the same amount of 2% alginate (Sigma). The mixed solution was dropped into 0.102 M CaCl_2_/0.15 M NaCl (pH 7.4) solution at the speed of 1.5 ml/min through a 23 G cannula equipped inside another 19 G cannula from which the air was ejected at the speed of 1.2 L/min. This procedure yielded alginate-beads containing HepG2 cells, whose diameters ranged from 300 to 500 μm. The alginate beads were washed with DMEM twice and cultured in DMEM supplemented with 10% FBS and 1% penicillin/streptomycin. Prior to the harvest of cells and medium, beads containing S-Hep were washed twice with DMEM and cultured in FBS-containing DMEM with or without TO for two days. Thereafter, the medium was exchanged with FBS-free medium containing the same concentration of TO, and the cells were incubated for another 24 hours. Then the media were collected, and the cells were dissolved in RIPA buffer after releasing them from alginate beads with the incubation in PBS containing 4 mM EGTA (pH 7.4) for 10 minutes.

### Quantification of Secreted Proteins in Medium

The concentrations of albumin, apoA-I and apoB in the media were measured by indirect sandwich enzyme-linked immunosorbent assay (ELISA) with human albumin ELISA quantification kit (Bethyl laboratories, Inc. Montgomery, TX.) and ELISA kits for human apoA-I and apoB (Mabtech Inc. Nacka Strand, Sweden). For the quantification of apoE levels, the media, the volumes of which were adjusted according to cell protein levels, were subjected to 10% SDS-PAGE followed by Western-blot analysis with anti-apoE antibody (Chemicon International Inc, Temecula, CA), and the intensities of the bands were measured by Image J (from the NIH).

### Preparation and Analysis of Nuclear Fraction

The nuclear fractions of HepG2 cells were obtained as follows: cells were dissolved in Buffer A (10 mM HEPES, 1.5 mM MgCl_2_, 10 mM KCl, 0.5 mM DTT, 0.05% NP40, protease inhibitor cocktail (Roche, Mannheim, Germany), pH7.9) and incubated on ice for 10 minutes, centrifuged at 900 g for 10 minutes. The pellets were homogenized in Buffer B (5 mM HEPES. 1.5 mM MgCl_2_, 0.2 M EDTA, 0.5 mM DTT, 26% glycerol, protease inhibitor cocktail, pH 7.9) supplemented with NaCl to the final concentration of 300 mM. Then, the solutions were centrifuged at 24,000 g for 20 minutes, and the supernatants were analyzed as the nuclear fractions of the cells. To quantify the levels of each nuclear protein, 30 μg of nuclear proteins extracted as above were subjected to 8% SDS-PGE followed by Western blot analyses with anti-Lamin A/C, anti-PPAR-α, anti-PPAR-γ, anti-RXR-α, anti-LXR-α, anti-HNF-1α, or anti-HNF-4α antibody (Santa Cruz Biotechnology).

### Quantitative Real Time PCR

Total RNAs extracted from M-Hep and S-Hep with GenElute mammalian total RNA miniprep kit (Sigma-Aldrich) were subjected to reverse transcription with Superscript II enzyme (Invitrogen Co. Carlsbad, CA). Real-time quantitative PCR was performed with LightCycler system (Roche Diagnostics Basel, Switzerland). The expression levels of the gene of interest were normalized to those of the endogenous control GAPDH mRNA, and the amounts of target gene expressions were expressed as a ratio to those of control cells. The following primers were used: for GAPDH, forward 5' CCACTCCTCCACCTTTGA 3' and reverse 5' GTGGTCCAGGGGTCTTAC 3'; for apoA-I, forward 5' TGTCCCAGTTTGAAGGCT 3' and reverse 5' ATCCTTGCTCATCTCCTGC 3'; for apoE, forward 5' GGGTCGCTTTTGGGATTAC 3' and reverse 5' CAACTCCTTCATGGTCTCG 3'; for ABCA1, forward 5' AAATCCATTGTGGCTGC 3' and reverse 5' GGGAGAGAGAGGTTGTGATAC 3'.

### FPLC Analysis

The media of S-Hep or M-Hep, the total volumes of which were 12 mL, were concentrated to about 500 μl by centrifugation through Amicon Ultara-15 (Millipore Co., Bedford, MA). Then 200 μl of concentrated medium was separated by FPLC utilizing Superose 6 column. The levels of apoB in the separated fractions were analysed with ELISA method. For the analyses of apoE and apoA-I, the separated fractions were subjected to Western blots utilizing anti-apoE antibody and anti-apoA-I antibody (Chemicon). To raise the sensitivity of western blot analysis, after the incubation with primary antibodies, the membranes were incubated in biotin-conjugated anti-goat IgG antibody (Sigma) and then detected by Vecstatin ABC kit (Vector laboratories, Inc, Burlingame, CA). FPLC fractions obtained from S-Hep with or without 2 μM TO were subjected to the measurement of triglycerides (TG) levels with enzymatic method (WAKO Pure Chemical Industries, Osaka, Japan). To standardize the TG values among samples from with or without TO, the values obtained were adjusted utilizing the TG levels of the media which was corrected with cellular protein levels.

### Statistical analysis

The results were expressed as mean ± SEM. Differences between two groups were evaluated with student's *t*-test, and the differences among more than assessed with one-way ANOVA, followed by multiple comparison tests. the *P *value less than 0.05 was deemed as statistically significant.

## List of Abbreviations

apoA-I: apolipoprotein A-I; apoB: apolipoprotein B; apoE: apolipoprotein E; TO: TO901317; M-Hep: HepG2 cells cultured in monolayer; S-Hep: HepG2 cells cultured in spheroidal form; HNF: hepatocyte nuclear factor; LXR: liver × receptor; PPAR: peroxisome proliferator-activated receptor; RXR: retinoid × receptor; ABC: ATP-binding cassette transporter; FPLC: fast protein liquid chromatography; FBS: fetal bovine serum; ELISA: enzyme-linked immunosorbent assay.

## Competing interests

The authors declare that they have no competing interests.

## Authors' contributions

MK participated in study design, carried out experiments and data analysis, and drafted the initial manuscript. NaI and MH participated in several experiments. NOI participated in the real-time PCR study. KM and KK were involved in study design and drafting manuscript. KT conceived of the study, coordinated the study design and helped to draft the manuscript. All authors read and approved the final manuscript.
